# Loss of FAM111B protease mutated in hereditary fibrosing poikiloderma negatively regulates telomere length

**DOI:** 10.3389/fcell.2023.1175069

**Published:** 2023-06-05

**Authors:** Maciej Kliszczak, Daniela Moralli, Julia D. Jankowska, Paulina Bryjka, Lamia Subha Meem, Tomas Goncalves, Svenja S. Hester, Roman Fischer, David Clynes, Catherine M. Green

**Affiliations:** ^1^ Nuffield Department of Medicine, Wellcome Centre for Human Genetics, University of Oxford, Oxford, United Kingdom; ^2^ Oncology Department, Weatherall Institute for Molecular Medicine, University of Oxford, Oxford, United Kingdom; ^3^ Nuffield Department of Medicine, Target Discovery Institute, University of Oxford, Oxford, United Kingdom; ^4^ Chinese Academy of Medical Sciences Oxford Institute, Oxford, United Kingdom

**Keywords:** FAM111B, POIKTMP, HFP, protease, DNA damage, telomeres, lung fibrosis

## Abstract

Hereditary fibrosing poikiloderma (HFP) is a rare human dominant negative disorder caused by mutations in the *FAM111B* gene that encodes a nuclear trypsin-like serine protease. HFP patients present with symptoms including skin abnormalities, tendon contractures, myopathy and lung fibrosis. We characterized the cellular roles of human FAM111B using U2OS and MCF7 cell lines and report here that the protease interacts with components of the nuclear pore complex. Loss of *FAM111B* expression resulted in abnormal nuclear shape and reduced telomeric DNA content suggesting that FAM111B protease is required for normal telomere length; we show that this function is independent of telomerase or recombination driven telomere extension. Even though *FAM111B*-deficient cells were proficient in DNA repair, they showed hallmarks of genomic instability such as increased levels of micronuclei and ultra-fine DNA bridges. When mutated as in HFP, FAM111B was more frequently localized to the nuclear envelope, suggesting that accumulation of the mutated protease at the nuclear periphery may drive the disease pathology.

## 1 Introduction

Hereditary fibrosing poikiloderma (HFP also known as POIKTMP, OMIM: 615704) is a rare dominant negative syndrome that affects multiple tissues throughout the body [reviewed in (Goussot et al., 2017; Arowolo et al., 2022; Hoeger, 2022; Macchiaiolo et al., 2022; Wu et al., 2022)]. Initial disease symptoms appear very early in childhood and include poikiloderma, photo-induced lesions, loss of facial hair and baldness. These phenotypes are accompanied by reduced sweating, heat intolerance and swelling of the extremities. Tendon contractures require medical attention to improve mobility of young HFP patients. As the affected individuals progress into adulthood other symptoms become more apparent. These include muscular wasting, digestive dysfunction (exocrine pancreatic insufficiency), respiratory obstruction (lung fibrosis) and pancreatic cancer. Furthermore, fibrotic lesions in the respiratory tract and skin can be detected in affected individuals (Mercier et al., 2013; Dereure, 2014; Mercier et al., 2015; Seo et al., 2016; Takeichi et al., 2017; Kazlouskaya et al., 2018; Sanchis-Borja et al., 2018; Chasseuil et al., 2019; Chen et al., 2019; Mercier et al., 2019; Zhang et al., 2019; Dokic et al., 2020; Leclerc-Mercier et al., 2022; Takimoto-Sato et al., 2022; Wu et al., 2022).

HFP is caused by mutation of a single allele of the *FAM111B* gene that is located on chromosome 11 (11q12.1). *FAM111B* encodes a 734aa protein with a trypsin-like protease domain at its C-terminus. It is 42% identical to the homologous serine protease FAM111A which is mutated in Kenney-Caffey and Gracile Bone Dysplasias, characterized by severe skeletal abnormalities (Unger et al., 2013). FAM111A localizes to DNA replication forks via a PIP-box mediated interaction with PCNA, where it processes DNA-protein crosslinks in response to replication stress (Kojima et al., 2020). Like FAM111A, the FAM111B protease has a conserved catalytic triad (residues H490, D544, S650) and shows protease activity *in vitro* (Hoffmann et al., 2020). Even though FAM111B does not contain a PIP box sequence, similarly to FAM111A it interacts with PCNA and the RFC complex (Hoffmann et al., 2020). FAM111B also possesses a centrally located FAM111B-specfic domain (112aa) of unknown function. The substrates and function of FAM111B, and thus the mechanism by which its mutation causes disease are currently unknown.

FAM111B is a nuclear protein expressed in tissue specific manner (brain, lungs, gastrointestinal tract, liver, pancreas, reproductive tissue, skin, bone marrow and lymphoid tissue) with peak cellular concentration during S phase of the cell cycle (Aviner et al., 2015; Sun et al., 2019; Kawasaki et al., 2020). Arrest of the cell cycle outside S phase leads to downregulation of FAM111B mRNA and protein levels (Yue et al., 2015; Cuella-Martin et al., 2016). Up until now, twelve different *FAM111B* disease causing mutations have been described [reviewed in (Goussot et al., 2017; Arowolo et al., 2022; Hoeger, 2022; Macchiaiolo et al., 2022; Wu et al., 2022)]. These affect single amino acids and cluster around two different regions of FAM111B, either in the protease domain (Y621D, T625N, T625P, R627G, R627S, S628G, S628N, S628R, F629C and F629S) or in the region of unknown function, very close to the FAM111B-specific domain (F416S, ΔK421 and Q430P). Given the autosomal dominant inheritance pattern of the disease, it is likely that these mutations result in a dominant negative or gain of function effect, perhaps by interfering with the wild type protein, that has particularly harmful consequences in cells of the connective tissue.

Telomeres are DNA sequences found at the end of chromosomes, which in humans consist of multiple TTAGGG repeats (Greider and Blackburn, 1985). The shortening of telomeres is a naturally occurring process that coincides with human aging, and re-expression of telomerase in human cells results in unlimited proliferation capacity (Bodnar et al., 1998). Short telomeres are recognized as DNA double strand breaks [reviewed in (de Lange, 2005)] and activate p53 pathway leading to apoptosis or senescence [reviewed in (Rossiello et al., 2022)]. Such an arrangement allows for a strict control of somatic cell’s proliferation capacity and suppresses duplication of cells with abnormally short telomeres [reviewed in (Hinds and Pietruska, 2017)]. Premature telomere shortening is detrimental for normal cellular function and has been linked to multiple human diseases including lung fibrosis [reviewed in (Armanios, 2022) and (Armanios et al., 2007; Tsakiri et al., 2007; Alder et al., 2008; Cronkhite et al., 2008; Diaz de Leon et al., 2010)].

Here we show that cells lacking FAM111B protease show hallmarks of genomic instability and have short and abnormal telomeres. Telomere lesions were induced in both telomerase-positive and negative cells strongly suggesting that FAM111B was not directly involved in either telomerase- or recombination-dependent telomere extension mechanisms. Interestingly, FAM111B protease could be detected at the nuclear periphery in the proximity of the nuclear envelope and interacted with components of the nuclear pore complexes. Mutant FAM111B localized more frequently to the nuclear lamina suggesting that accumulation of FAM111B at the nuclear periphery might drive disease pathology.

## 2 Results

### 2.1 FAM111B protease localizes to the nuclear periphery and interacts with nuclear pore complexes

**FIGURE 1:**
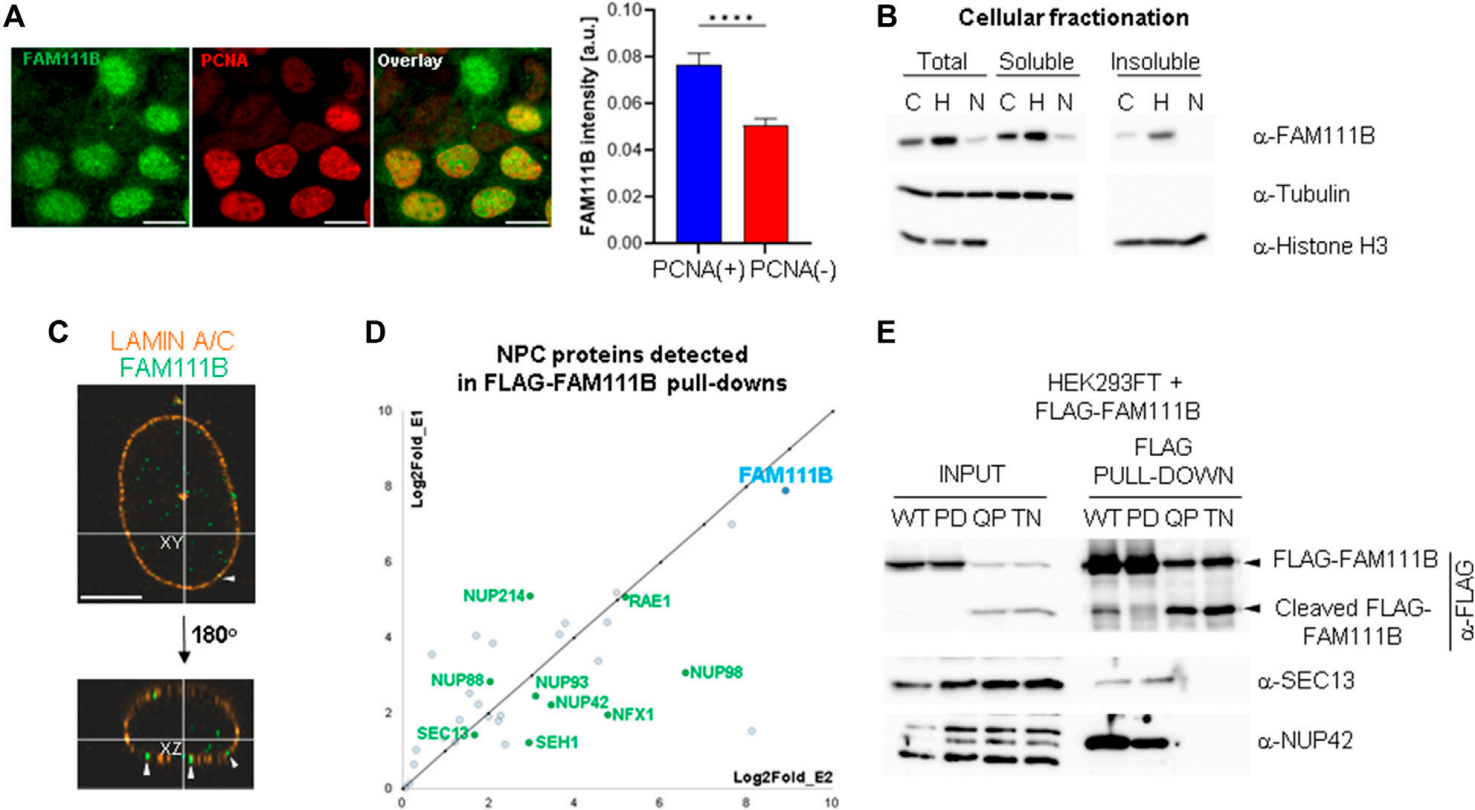
FAM111B localizes to the nuclear lamina where it interacts with nuclear pore complexes. **(A)** Widefield images showing non-detergent extracted FAM111B and PCNA staining in U2OS cells (left panels, scale bar 10 μm) and quantification of FAM111B staining intensity in PCNA positive and negative cells (*N* = 3, ∼400 cells per experiment, OWA test, error bars show S.E.M.). **(B)** Immuno-blots of different cellular fractions of U2OS cells before and after treatment with cell cycle inhibitors hydroxyurea (H, S-phase arrest) and Nutlin-3a (N, G1-and G2/M-phase arrests). **(C)** Confocal images of detergent extracted U2OS cells stained with FAM111B (green) and Lamin A/C (orange) antibodies, white arrow heads indicate FAM111B embedded in the nuclear envelope. Scale bar 5 μm. **(D)** FAM111B interactions with nuclear pore complex components detected by mass spectrometry. The chart shows log2fold change in peptide abundance over FLAG control from two separate biological repeats. **(E)** Immuno-blots confirm mass spectrometry data showing complex formation between FLAG-FAM111B and nucleoporins (SEC13 and NUP42) in HEK293T cells.

Staining of human cells with FAM111B antibodies revealed that the protease can be found in both cytoplasm and nucleus; however, the majority of FAM111B resided in the latter compartment (but not in nucleoli) (Figure 1A). The modulation of FAM111B protein levels, dependent on cell cycle position (enriched in S-phase cells with characteristic PCNA replication foci) was very apparent and consistent with previously published reports (Sun et al., 2019; Hoffmann et al., 2020; Kawasaki et al., 2020) (Figure 1A). Fractionation of U2OS cells showed that FAM111B was largely soluble, with a fraction of the protease also bound to insoluble components of the cell such as chromatin or cellular membranes, particularly after blocking the cell cycle in early S-phase with hydroxyurea (Figure 1B). To determine whether the immobilized fraction of the FAM111B accumulated in a particular nuclear compartment, we analyzed FAM111B staining in U2OS cells using high resolution microscopy. Prior to analysis, soluble and proteins weakly bound to the structural components of the cell (such as cytoskeleton, membranes or chromatin, etc.) were removed by detergent extraction and the remaining cellular material stained with antibodies against FAM111B and Lamin A/C. FAM111B staining in these conditions revealed a clear three-dimensional organization inside the nucleus (Figure 1C and Suppelementary Movie S1A), again no nucleolar staining was observed. Interestingly, FAM111B also localized to the nuclear periphery where it either interacted with or was found embedded in the nuclear lamina (Figure 1C, white arrow heads, Suppelementary Movies S1A, S1B). This suggested that FAM111B interacted with the lamin network. To test whether FAM111B directly interacted with lamin proteins, we characterized FAM111B binding partners using mass spectrometry. Analysis of the FAM111B interactome revealed that FAM111B did not form complexes with lamins but instead with components of the nuclear pore complexes (NPCs) (Figure 1D and Supplementary Figure S1A). Co-immunoprecipitation experiments with FLAG-FAM111B confirmed that the protease interacted with two nucleoporins SEC13 and NUP42 (Figure 1E). This was consistent with the observed localization of FAM111B and suggested that FAM111B may be recruited to the nuclear periphery through the interaction with components of the NPCs.

### 2.2 Different variants of FAM111B, including HFP mutations cause accumulation of the protease at the nuclear periphery

**FIGURE 2:**
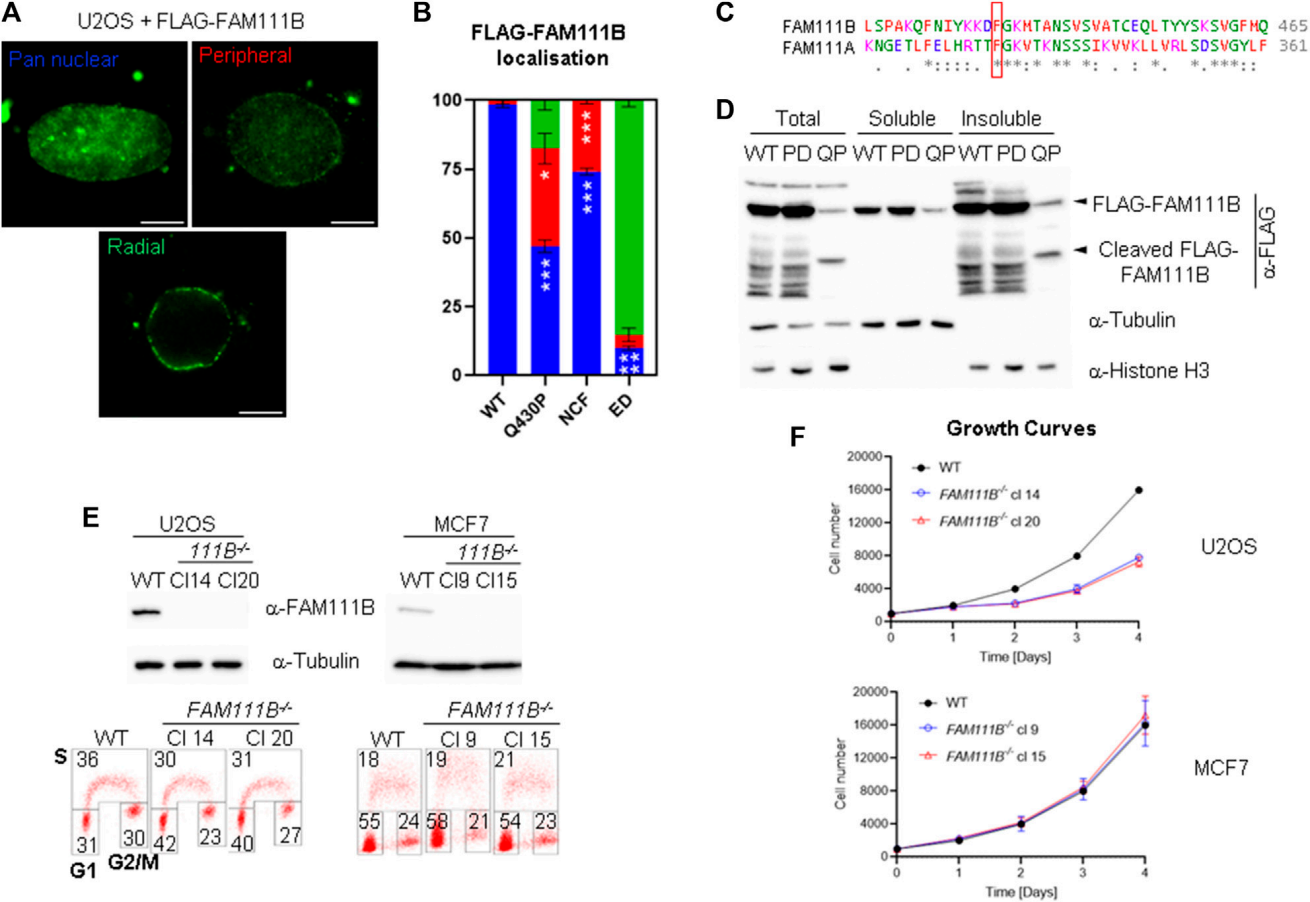
Mutant FAM111B accumulates at the nuclear periphery. **(A)** Widefield images of detergent extracted cells showing three types of staining patterns quantified in cells overexpressing FLAG-FAM111B variants: wild-type (WT), Q430P (HFP), N-terminal cleavage fragment (NCF, 1-438aa) and ED (Δ279-386). Scale bar 5 μm. **(B)** Quantification of FLAG-FAM111B staining patterns from **(A)**. Blue—pan nuclear, Red—peripheral enriched, Green—exclusively peripheral. Bars show averages of *N* = 3 experiments. In each at least 50 cells per sample was scored and analyzed using OWA test. **(C)** ClustalW sequence alignment of FAM111B auto-cleavage site from human FAM111 proteases. The conserved cleavage site F438 in FAM111B and F334 in FAM111A are indicated with a red frame. **(D)** Immuno-blots of cellular fractions of U2OS cells over-expressing FLAG-tagged FAM111B wild-type (WT), protease dead (PD) and HFP mutant (Q430P, QP). **(E)** Immuno-blots of FAM111B levels in wild-type (WT) and *FAM111B* ^*−/-*^ clones 14 and 20 (U2OS) and clones 9 and 15 (MCF7) (top panels). Cell cycle distribution was measured using BrdU staining and 2D flow cytometry. Number in the corners of the boxes indicate % of the cells in S, G1 and G2/M phases of the cell cycle (bottom panels). **(F)** Growth curves of U2OS (clones 14 and 20) and MCF7 (clones 9 and 15) *FAM111B*-deficient cells. Results show averages of *N* = 3 experiments, error bars show S.E.M.

Localization of the endogenous FAM111B protease at the nuclear periphery and its interactions with the nuclear pore complexes suggested that this localization may be important for FAM111B function. We wondered whether different variants of FAM111B including mutation found in the HFP syndrome could affect this localization. We over-expressed different FAM111B variants as FLAG-fusions in U2OS cells, extracted the soluble proteins with detergent and stained the cells with FAM111B antibodies (Figure 2A). We have observed three distinct staining patterns which we quantified as: pan nuclear, peripheral enriched and exclusively peripheral (Figure 2A). FLAG-tagged FAM111B wild-type (WT) showed similar staining pattern to the endogenous FAM111B (Figure 2A, pan nuclear and B), whereas in the cells over-expressing the HFP variant (Q430P) we could observe a small fraction of the cells where FLAG-FAM111B markedly accumulated at the nuclear periphery (Figure 2A, peripheral enriched and B). Because the HFP mutant variants of FAM111B undergo auto-cleavage into proteolytic fragments (Figures 1E, 2D), which might be relevant to their dominant negative function and disease development (Hoffmann et al., 2020), we investigated whether the resulting fragments had similar potential to localize to the nuclear periphery. The auto-cleavage site in the FAM111A protease (F334) is conserved in FAM111B (F438, Figure 2C) (Kojima et al., 2020), and the theoretical molecular weight of the N-terminal fragment after cleavage at this site corresponded to the actual mass detected by immunoblotting (Figures 1E, 2D). We cloned both WT predicted fragments: N-terminal cleavage fragment (NCF) and C-terminal cleavage fragment (CCF) and over-expressed them in U2OS cells as FLAG-fusion proteins. The NCF fragment showed a similar localization to the HFP mutation with partial accumulation at the periphery (Figure 2A, peripheral enriched and B) whereas the C-terminal fragment did not express to detectable levels. Cellular fractionation of the cells over-expressing FAM111B with the disease relevant Q430P (QP) mutation showed that the N-terminal auto-cleavage fragment accumulated exclusively in the insoluble fraction of the cell (Figure 2D). Furthermore, we observed that FAM111B lacking a centrally located domain which is not conserved between FAM111A and FAM111B (FAM111B extra domain, Δ279-386aa, ED) demonstrated exclusive accumulation of the FAM111B protease at the nuclear periphery (Figure 2A, exclusively peripheral, and B). Our analysis suggested that FAM111B activity might be transiently required at the nuclear periphery and that accumulation of HFP associated Q430P mutation or truncated forms of FAM111B at this location could be potentially pathological.

### 2.3 FAM111B-deficient cells have abnormal nuclear morphology

**FIGURE 3:**
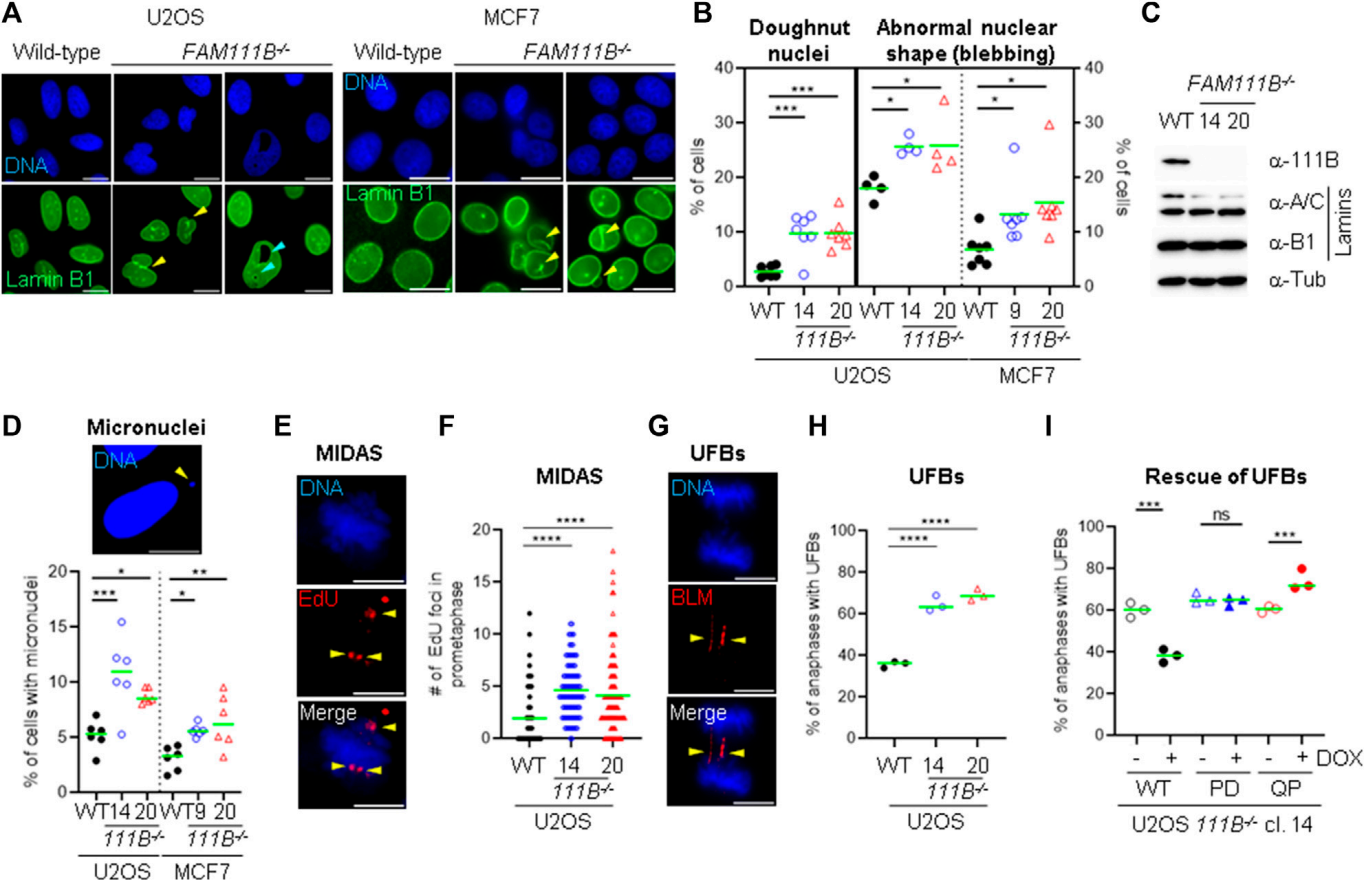
Loss of FAM111B induces abnormal nuclear morphology and spontaneous DNA damage. **(A)** Widefield images showing Lamin B1 staining in U2OS and MCF7 wild-type (WT), and *FAM111B*-deficinet cells. Nuclear blebbing and doughnut nuclei are indicated with yellow and magenta arrow heads, respectively. Scale bar 10 μm. **(B)** Quantification of nuclear abnormalities in U2OS (clone 14 and 20) and MCF7 (clone 9 and 15) *FAM111B* knockout cells. Green bars show averages of *N* = 7 experiments, with at least 180 nuclei analyzed per repeat. **(C)** Immuno-blots showing normal levels of lamin proteins in the U2OS cells lacking FAM111B. **(D)** Widefield image of micronuclei (scale bar 10 μm) and their quantification in U2OS (clone 14 and 20) and MCF7 (clone 9 and 15) *FAM111B*-deficient cells. Green bars show averages of *N* = 7 experiments, with at least 180 cells scored per sample in each experiment. **(E)** Widefield images of MIDAS, detected by the incorporation of EdU during prometaphase. Scale bar 5 μm. **(F)** Quantification of MIDAS in *FAM111B* knockout cells. Green bars show averages of *N* = 3 experiments, with at least 50 prometaphases scored per sample in each experiment. **(G)** Widefield images of ultra-fine DNA bridges. Scale bar 5 μm. **(H)** Quantification of ultra-fine DNA bridges in anaphase cells. Green bars show averages of *N* = 3 experiments, with at least 50 anaphases scored per sample in each experiment. **(I)** Quantification of ultra-fine DNA bridges in *FAM111B* knockout and cell line (clone 14) rescued with either FAM111B wild-type (WT), protease dead (H490A, PD) or HFP mutant (Q430P, QP). Green bars show averages of *N* = 3 experiments, where in all samples but Q430P + DOX, 50 anaphases were scored per sample in each experiment (Q430P had total of 61 anaphases in *N* = 3). OWA test was used in B, D, H and I, whereas K-W test in **(F)**.

Because FAM111B localized to the nuclear lamina we predicted that loss of FAM111B may have a negative effect on nuclear morphology. To test this hypothesis, we employed a reverse genetic approach and manipulated the *FAM111B* gene in human cells (U2OS and MCF7) using Cas9-mediated editing and two independent guide RNAs (Figure 2E, top panel). Because CRISPR-Cas9 engineering can generate chromosomal rearrangements (Rayner et al., 2019), we screened *FAM111B* locus integrity in the targeted clones using fluorescent *in situ* hybridization (FISH) probes, and selected those which had a karyotype identical to wild-type cells and the intact probe pattern at the targeted *FAM111B* loci [Supplementary Figure S1B, analogous data for MCF7 clones (not shown)]. As previously reported (Blomen et al., 2015; Hart et al., 2015; Wang et al., 2015; Hoffmann et al., 2020), loss of FAM111B expression was compatible with cellular viability; however, U2OS cells lacking FAM111B showed approximately 2-fold growth defect characterized by accumulation of cells in G1 phase of the cell cycle (Figure 2E bottom panel and F). Staining of *FAM111B* ^*−/-*^ cells with antibodies against lamins showed that the *FAM111B*-deficient cells had increased levels of nuclear abnormalities such as abnormal shape (including nuclear blebbing) and doughnut nuclei (Figures 3A, B), suggesting that FAM111B function at the nuclear lamina may be required for the maintenance of normal nuclear shape. Similar phenotypes are observed in cells with lamin mutations or other genetic backgrounds caused by deficiencies in the components of the lamina network [reviewed in (Dobrzynska et al., 2016)]. We tested whether loss of FAM111B protease expression had any effect on the stability of lamin proteins. We found normal levels of Lamin B1 and only a small change in the Lamin A to C ratio in U2OS cells lacking FAM111B (Figure 3C), suggesting that the protease is unlikely involved in direct processing or regulation of lamin stability.

### 2.4 FAM111B is dispensable for DNA repair but loss of *FAM111B* in U2OS cells increased levels of endogenous DNA damage

The association of FAM111B with nuclear pore complexes suggested a potential role of the protease in the nuclear transport. We sequenced mRNAs isolated from nuclear and cytoplasmic fractions of wild-type and *FAM111B* KO cells and found no major differences in the cellular mRNA content in the absence of FAM111B (data not shown). This suggested that global mRNA transport is not affected by the loss of *FAM111B* expression; however, we cannot rule out the possibility that transport of specific mRNAs or proteins may be affected in the absence of FAM111B. Besides NPCs canonical function as pores, they also serve as hubs for recruitment of chromatin and nuclear factors required for RNA synthesis, DNA repair and telomere maintenance [reviewed in (Lamm et al., 2021)]. Nuclear pore complexes participate in maintenance of genome stability through the recruitment of DNA repair intermediates such as double strand breaks and collapsed replication forks (Khadaroo et al., 2009; Whalen et al., 2020). Therefore, we wondered whether similarly to nuclear pore complex mutants (Palancade et al., 2007; Gaillard et al., 2019; Kramarz et al., 2020), cells lacking *FAM111B* could be DNA repair deficient. We treated wild-type and mutant cells with compounds that induce DNA damage and with compounds that interfere with cell cycle, DNA replication or transcription. We analyzed survival of wild-type and mutant cells using a resazurin viability assay. Because cell survival experiments rely on proliferation and measurement of relative metabolic activity of the cells and U2OS *FAM111B* ^*−/-*^ mutants have a growth defect compared to WT, we focused only on MCF7 *FAM111B*-null cells, which we found to be no more sensitive than their wild-type counterparts to multiple drugs tested (Supplementary Figures S2A–J). This indicated that FAM111B was not directly involved in DNA repair, replication or transcription in MCF7 cells. Even though *FAM111B* ^*−/-*^ cells were proficient in their responses to DNA damage, we wondered whether their abnormal nuclear shape was associated with increased levels of endogenous DNA damage as is observed in mutants of the components of the nuclear lamina (Gonzalez-Suarez et al., 2011; Kreienkamp et al., 2016; Earle et al., 2020). We thus screened the mutant cells for hallmarks of genomic instability. Quantification of micronuclei in the mutant cells (Figure 3D) showed greater levels of these events in the absence of FAM111B protease compared to wild-type cells. Consistently, we could also observe higher frequencies of events upstream of micronuclei formation such as increased mitotic DNA synthesis (MIDAS) foci (Figures 3E, F) and greater levels of BLM-coated DNA ultra-fine bridges (UFBs) in U2OS cells (Figures 3G, H). Re-introduction of wild-type but not protease dead (PD) variant into the *FAM111B* ^*−/-*^ cells could rescue the normal levels of UFBs (Figure 3I and Supplementary Figure S2L). This confirmed that the observed phenotype is specific to the loss of FAM111B and that protease activity of FAM111B plays an essential role in maintaining low levels of BLM bridges in U2OS cells. In the same assay, the disease Q430P variant did not restore WT levels, suggesting that HFP Q430P mutation is at least partially defective in this function (Figure 3I and Supplementary Figure S2L). We conclude that *FAM111B*-deficient cells, even though proficient in DNA repair accumulate DNA damage and show hallmarks of genomic instability.

### 2.5 Cells lacking FAM111B have short and abnormal telomeres

**FIGURE 4:**
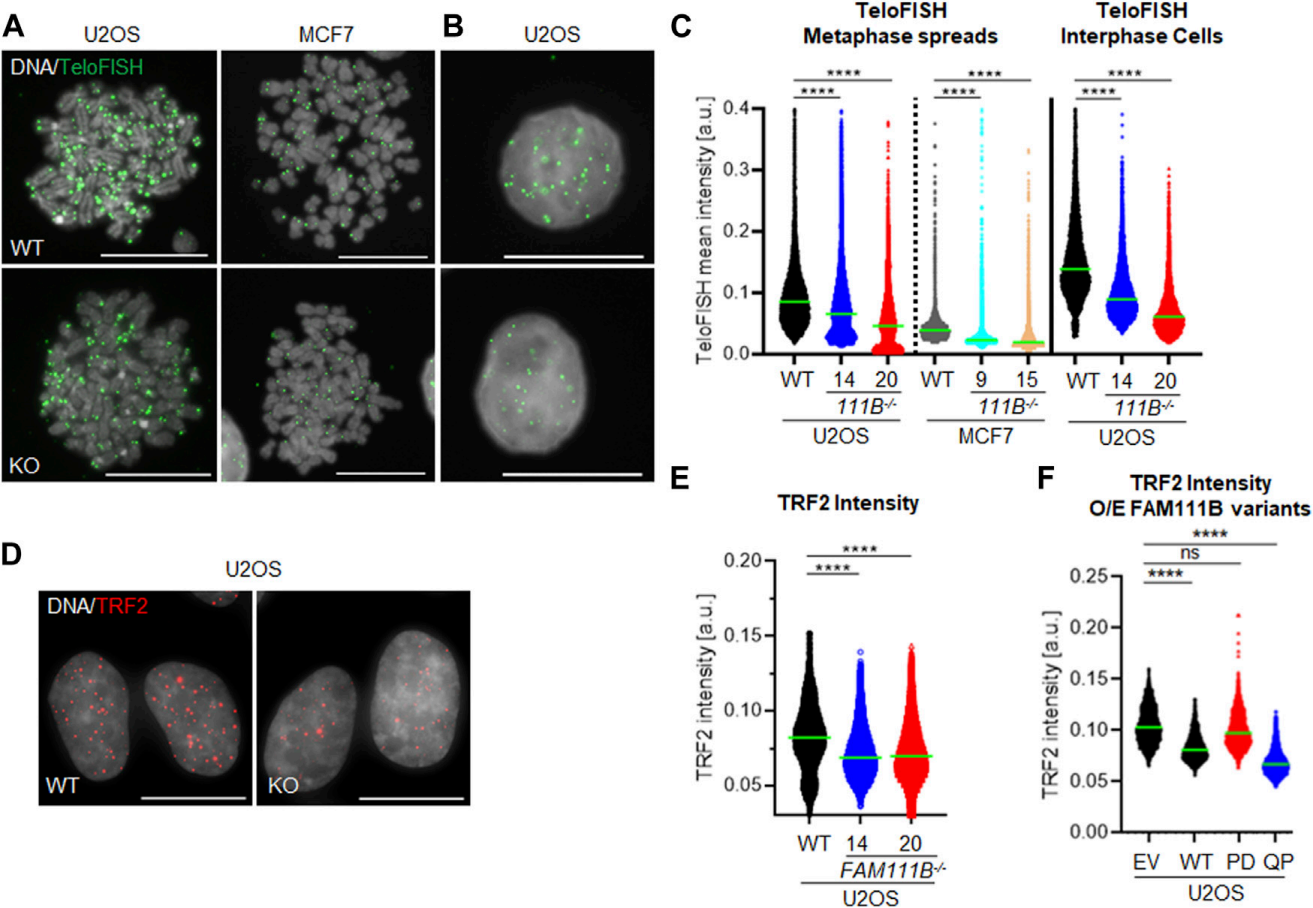
*FAM111B*-deficient cells have shorter telomeres. **(A)** Widefield images of chromosome spreads and **(B)** interphase cells from wild-type (WT) and *FAM111B* knockout cells stained with fluorescent probe against telomeric repeats. Scale bar 20 μm. **(C)** Quantification of individual TeloFISH foci intensity in metaphase spreads and interphase cells. Green bars show averages of *N* = 3, with at least 30 metaphase spreads or 60 cells scored per sample in each experiment. **(D)** Widefield images of wild-type (WT) and *FAM111B* ^*−/-*^ U2OS cells stained with TRF2 antibodies. Scale bar 20 μm. **(E)** Quantification of TRF2 foci intensity in U2OS wild-type (WT) *FAM111B* negative U2OS cells. Green bars show averages of *N* = 3 experiments, where 400 cells was scored per sample in each experiment. **(F)** Quantification of TRF2 intensities in U2OS cells over-expressing empty vector (EV) or FLAG-tagged FAM111B variants wild-type (WT), protease-dead (PD) or HFP mutant (Q430P, QP), respectively. Green bars show averages of *N* = 3 experiments, with at least 50 cells per sample scored in each experiment. K-W test was used in C, E and **(F)**.

**FIGURE 5:**
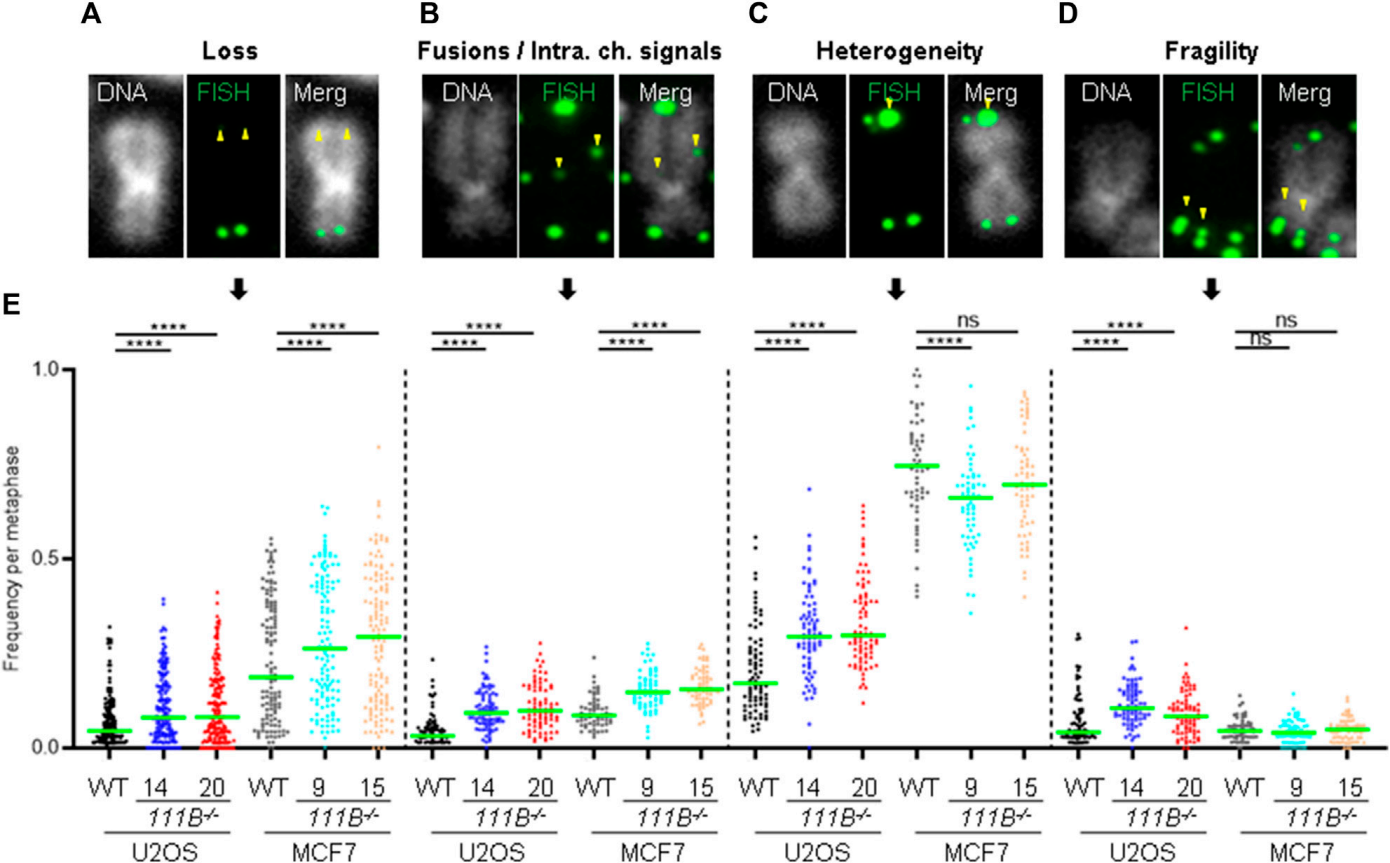
Loss of *FAM111B* induces telomere abnormalities. **(A–D)** Widefield images of individual chromosomes stained with a fluorescent probe against telomeric repeats, showing telomeric abnormalities detected in *FAM111B*-deficient cells. These include telomere loss, fusions/intrachromosomal telomere signals, heterogeneity and fragility. **(E)** Quantification of telomeric phenotypes in U2OS and MCF7 wild-type (WT) and *FAM111B* ^*−/-*^ cells per metaphase. Green bars show averages of *N* = 3 experiments, with at least 30 metaphase spreads scored per sample in each experiment. Only MCF7 telomer heterogeneity data was analyzed with OWA test, rest of the datasets were analyzed with K-W test.

Beside the role that the nuclear pore complexes play in the responses to DNA damage they are also involved in the telomere lengthening and repair by recruiting telomeres to the nuclear envelope (Khadaroo et al., 2009). Interestingly, HFP patients develop pulmonary fibrosis which strongly correlates with short telomeres [reviewed in (Goussot et al., 2017; Arowolo et al., 2022; Hoeger, 2022; Macchiaiolo et al., 2022; Wu et al., 2022) and (Armanios, 2022)]. To test whether FAM111B may be required for telomere homeostasis, we looked at the length and condition of the telomeres in *FAM111B* ^*−/-*^ cells. Quantification of the telomeric FISH probe intensity in the metaphase spreads prepared from wild-type and mutant cells showed that cells lacking FAM111B protease indeed had greatly reduced telomeric DNA content (Figures 4A, C), and a similar phenotype in interphase cells was also observed (Figures 4B, C). Consistently, shorter telomeres in *FAM111B* ^*−/-*^ U2OS cells recruited less of the shelterin component TRF2 compared to wild-type counterparts (Figures 4D, E). These phenotypes were independent of passage number (Supplementary Figure S1D) and were not caused by off target effect of the CRISPR deletion method applied (CRISPR-Cas9 treated clones with wild-type like FAM111B levels have normal levels of TRF2 foci intensities) (Supplementary Figure S1C). Because pulmonary fibrosis observed in the FAM111B mutation carriers can be directly linked to short telomeres, we tested whether over-expression of HFP variant Q430P in U2OS cells had any effect on telomeres. Telomere shortening requires multiple rounds of division to be detectable and unfortunately, prolonged over-expression of FAM111B (wild-type or mutant but not protease dead (Hoffmann et al., 2020), and our unpublished data) is toxic, preventing direct measurement of telomere length in extended experimental conditions. Therefore, we transiently over-expressed different variants of FAM111B in U2OS cells for no longer than 24 h and instead quantified TRF2 recruitment to the telomeres. Interestingly, overexpression of either the HFP mutant, or the wild-type protein but not the protease dead variant of FAM111B induced a rapid loss of TRF2 intensity (Figure 4F). The fact that this is detected in conditions when the telomere shortening cannot yet be observed suggests that TRF2 loss may drive telomere shortening in HFP. The fact that we see the same TRF2 reduction upon overexpression of the WT protein also implies that the normal cell cycle regulation of FAM111B, as well as the function impaired by the HFP mutation, is likely necessary for proper telomere function. Because only critically short telomeres are detrimental for cell viability and genome integrity (Hemann et al., 2001), we looked whether *FAM111B* ^*−/-*^ cells had any telomeric abnormalities beside short length. The same metaphase spread samples were used to determine levels of four phenotypes (Figures 5A–D, indicated by yellow arrowheads): loss of telomere signal, telomere fusion/intrachromosomal telomere signal, telomere heterogeneity and fragility. Both cell lines in the absence of FAM111B protease showed increased levels of telomere loss and fusion/intrachromosomal telomere signal events (Figures 5A, B, E), whereas telomeric heterogeneity and fragility was specifically increased only in U2OS cells (MCF7 on average showed slight decrease, Figures 5C–E). Loss of *FAM111B* induced telomere shortening both in telomerase positive MCF7 cells and in U2OS cells which utilize alternative lengthening of telomeres (ALT), strongly indicating that FAM111B operates independently of these telomere lengthening mechanisms. Consistent with this, levels of C-circles in the *FAM111B*-deficient U2OS cells were comparable to wild-type (Supplementary Figure S1E) and MCF7 knockout cells showed wild-type like survival to telomere replication inhibitor TMPyP4 (Supplementary Figure S2K).

## 3 Discussion

Our analysis of FAM111B cellular functions showed that the protease is a non-essential, nuclear protein regulated by the cell cycle (Figures 1A, B). We discovered that a subpopulation of FAM111B localizes to the nuclear periphery where it interacts with nuclear pore complexes (Figures 1C, D) but not with the components of the nuclear lamina such as lamins or emerin (Figure 1D and Supplementary Figure S1A). In particular, the FAM111B interactome contains proteins rich in Phe-Gly (FG) repeats: NUP98, NUP42 and NUP214. While we did not identify whether these are the FAM111B protease targets but we note that an FG containing protein, NUP62 is a target of the homologous protein FAM111A (Nie et al., 2021). Mutant FAM111B is more frequently localized to the nuclear periphery and disease relevant variants accumulate in the insoluble fraction of the cell (Figures 2A, B, D). Although we did not detect any robust differences in the mRNA content of the FAM111B knockouts compared to WT we note that by mass spectrometry FAM111B interacts with RAE1 and GLE1, components of the RNA export machinery, suggestive of a potential role for FAM111B in this function. Loss of FAM111B induces dramatic nuclear shape abnormalities (Figures 3A, B) and genomic instability (micronuclei, MIDAS and UFBs) (Figures 3D–I). Interestingly, FAM111B is not required for normal responses to DNA damage or inhibition of DNA replication/transcription (Supplementary Figures S2A–J) but is needed to maintain normal telomere length in both telomerase- and ALT-positive cells. In the absence of FAM111B protease, telomeres are shorter and recruit less of the shelterin component TRF2 (Figures 4A–E). Many chromosomes in *FAM111B*-deficient cells showed no detectable telomeric DNA and frequently engaged in fusion events (Figures 5A–E). This suggests that loss of FAM111B causes the formation of short telomeres, via a mechanism that potentially involves the loss of TRF2. Importantly, overexpression of the HFP mutant Q430P also leads to loss of TRF2, suggesting that this may be the mechanism that drives the short telomere phenotype seen in this disease.

The FAM111B paralog protease FAM111A interacts with PCNA via a PIP-box motif and is required for removal of DNA-protein crosslinks (Kojima et al., 2020). In our hands *FAM111B*-deficient cells were no more sensitive to cis-platin, camptothecin, etoposide and formaldehyde that, besides damaging DNA, also induce protein-DNA crosslinks (Supplementary Figures S2D–F, J). This suggests that any role of FAM111B at chromatin may be different from FAM111A. 3D Genome organization is dependent on robust interactions with the nuclear lamina and disruption of the nuclear envelope can inhibit DNA synthesis (Spann et al., 1997; Moir et al., 2000). Because FAM111 proteases interact with components of the nuclear pore complexes [this manuscript and (Hoffmann et al., 2020; Nie et al., 2021)], FAM111 proteases may influence DNA replication and transcription by targeting proteins at this specific nuclear localization. Currently no substrate of FAM111B has been identified but at least one component of the nuclear pore complex (NUP62) has been recently described as a target of FAM111A (Nie et al., 2021). Because of the many similarities between the two proteases, we hypothesize that FAM111B likely also targets components of the NPC. In our current working model for FAM111B function this protease activity is needed in a properly controlled fashion at NPCs to maintain normal telomeric structures. In the absence of FAM111B, or the presence of HFP-causing mutations, critically short telomeres driven by the loss of TRF2, induce DNA synthesis in mitosis (MIDAS), BLM bridges and micronuclei formation in the following G1 phase of the cell cycle (Figures 2D–I). Consistent with this model, lymphoblasts isolated from HFP patients showed spontaneous chromosome aberrations (Roversi et al., 2021) and HFP symptoms (e.g., poikiloderma and sparse hair) are also present in DNA repair and premature aging syndrome Rothmund-Thomson with defective telomeres (reviewed in [(Luong and Bernstein, 2021)]. The cellular phenotypes described in this manuscript could explain why patients carrying FAM111B mutations suffer from pulmonary dysfunction as fibrosis of the lungs strongly correlates with short telomeres (Armanios et al., 2007; Tsakiri et al., 2007; Alder et al., 2008; Cronkhite et al., 2008; Diaz de Leon et al., 2010). Interestingly, mutant FAM111B proteins including HFP associated mutations showed more prominent localization to the nuclear periphery (Figures 2A, B) suggesting a potential mechanism of the disease pathology where deregulated FAM111B-dependent processing of yet unidentified target(s) at the proximity of nuclear pore complexes causes shorter telomeres. This in turn precipitates spontaneous DNA damage and affects cellular fitness resulting in threshold-specific deterioration of tissues such lungs, skin, muscles and tendons.

## 4 Materials and methods

### 4.1 Cell culture

Human osteosarcoma (U2OS), human breast cancer (MCF7) and human embryonic kidney cells (HEK293T) cells were cultured in Dulbeco’s Modified Eagle Medium (DMEM, Sigma) supplemented with 10% fetal bovine serum (Sigma), 1% glutamate (Sigma) and 1% penicillin/streptomycin (Sigma) at 37°C in humidified 5% CO_2_ atmosphere. For conditional protein expression 1 μg/mL of doxycycline (Sigma) was added to the culture media and incubated for up to 24 h.

### 4.2 Gene and CRISPR construct cloning

All gene cloning was performed using Gateway^®^ system (Invitrogen) unless stated otherwise. Briefly, human FAM111B cDNA was amplified using high fidelity Q5 DNA polymerase (New England Biolabs), cDNA template (kind gift from Dr Nicola Burgess-Brown and Dr Alejandra Fernandez-Cid) and cloned into pDONR-ZEO (Invitrogen) vector to obtain pDONR-ZEO-FAM111B-WT (wild-type). Protease dead (H490A), patient mutations (Q430P and T625N) and ΔED (Δ279-386) variants were constructed by site directed mutagenesis using pDONR-ZEO-FAM111B-WT as a template and Q5 polymerase. FAM111B cDNAs were then transferred from pDONR-ZEO to pHAGE-(N)-HA-FLAG-(PURO) vector for constitutive expression or to pSIN3A-(C)-HA-(PURO) vector for conditional expression (TET^ON^) (kind gift from Prof J. Ross Chapman). *FAM111B* targeting constructs were cloned into pX330-(PURO) (kind gift from Prof J. Ross Chapman) using previously published protocol (Ran et al., 2013). For primer details please refer to Supplementary Material.

### 4.3 Antibodies

Primary antibodies were as follows: mouse anti-human BLM (sc-365753, Santa Cruz Biotechnology) at 1:250 (IF), mouse anti-BrdU (347580, Becton Dickinson) at 1:20 (Flow cytometry), rabbit anti-human FAM111B (HPA038637, Atlas Antibodies) at 1:1,000 (IB and IF), mouse anti-human Histone H3 (10799, Abcam) at 1:1,000 (IB), mouse anti-human Lamin A/C (sc-376248, Santa Cruz Biotechnology) at 1:1,000 (IB and IF), rabbit anti-human Lamin B1 (12987-1-AP, Proteintech Group) at 1:1,000 (IB and IF), rabbit anti-human NUP42 (16587-1-AP, Proteintech Group) at 1:1,000 (IB), mouse anti-human PCNA (sc-56, Santa Cruz Biotechnology) at 1:1,000 (IF), rabbit anti-human SEC13 (15397-1-AP, Proteintech Group) at 1:1,000 (IB), mouse anti-human TRF2 (NB100-56506, Novus Biologicals) at 1:1,000 (IF), mouse anti-human αTubulin (T6074, Sigma) at 1:10,000 (WB) or 1:1,000 (IF). Secondary antibodies were as follows: goat anti-rabbit Alexa Fluor 488 (A32731, ThermoFisher Scientific) at 1:1,000 (IF), donkey anti-mouse Alexa Fluor 555 (A32773, ThermoFisher Scientific) at 1:1,000 (IF), goat anti-rabbit HRP (31430, ThermoFisher Scientific) at 1:25,000 (IB) and donkey anti-mouse HRP (31458, ThermoFisher Scientific) at 1:25,000 (IB).

### 4.4 Microscopy and immuno-fluorescence

U2OS or MCF7 cells were seeded at 2 × 10^5^ onto glass cover slips in 6 well plates. 48 h post-seeding cells were fixed with 4% formaldehyde in PBS for 10 min at room temperature, washed three times with PBS (3 min each), permeabilized with CSK buffer for 5 min at room temperature (10 mM PIPES-KOH pH6.8, 100 mM NaCl, 300 mM sucrose, 1 mM EGTA, 1 mM MgCl2, 1 mM DTT and 0.5% Triton X-100) supplemented with protease and phosphatase inhibitors and again washed three times with PBS (3 min each). For detergent extraction experiments, cells were first exposed to CSK buffer for 5 min at room temperature washed three times with PBS (3 min each) and then fixed with 4% formaldehyde for 10 min at room temperature, followed by washes with PBS (3 min each). Cover slips were then blocked with 3% BSA/PBS for 30 min at room temperature, incubated with primary antibodies in the blocking buffer for 1 h at room temperature and washed three times with PBS (3 min each). This was followed by the incubation of cover slips in secondary antibodies diluted in the blocking buffer for 1 h at room temperature, three washes with PBS and mounting the cover slips onto glass slides with Vectashield + DAPI (H-1200-10, 2BeScientific). For staining of ultra-fine DNA bridges (UFBs), 2 × 10^5^ cells was seeded onto cover slips and 24 h later cells were exposed to 2 mM thymidine for 16 h. The next day, the nucleotide was washed away (three times with warm media) to release cells from the block and trapped in pro-metaphase for 3 h at 9 h post-thymidine removal with the addition of nocodazole at 200 ng/mL. Microtubule poison was then washed away (three times with warm fresh media) and cells harvested 30–45 min after release from the nocodazole block. For the staining of UFBs in the FAM111B rescue cell lines, doxycycline was added exactly 24 h prior to 30 min release mark from the nocodazole block. For detection of EdU foci in prometaphase cells (MIDAS), U2OS cells were seeded the same way as in for analysis of UFBs, however; cells were not released from the nocodazole block, labelled for last 30 min with 10 μM EdU and fixed with 4% formaldehyde for 10 min at room temperature. EdU detection was performed according to the manufacturer’s instructions (Invitrogen). Analysis was performed using wide-field Leica DM4000 or AiryScan 2 (Zeiss) microscopes.

### 4.5 Cell transfections

For transient transfections of U2OS or HEK293T cells, 70%–80% confluent cultures in 6 well plates or 10 cm dishes were transfected using 3 µg or 18 µg of linear PEI (Sigma), 0.2 mL or 1 mL OPTI-MEM medium (Life Technologies) and 1 µg or 6 µg of DNA, respectively. Cells were analyzed by microscopy or harvested 24 h post-transfection. To obtain stably expressing U2OS clones, 70%–80% confluent cultures in 10 cm dishes were transfected as above and 24 h post-transfection cells were exposed to Puromycin (Invitrogen) at 0.5 μg/mL. After approximately 2-3 weeks, single colonies were collected by trypsinization and expanded.

### 4.6 Generation of *FAM111B*-deficient clones

As described in the section “Cell transfections,” pX330-(PURO)-*FAM111B* gRNA #1 and #2 constructs were delivered to U2OS and MCF7 by transfections with linear PEI in 10 cm dishes. After 24 h post-transfection, cells were pulsed with puromycin at 1 μg/mL for 72 h. Approximately 2-3 weeks later, single colonies were collected by trypsinization and expanded in 24 and then 6 well plates. When clones reached 90% confluency in 6 well plates, half of the cultures was frozen, and the remaining cells harvested and analyzed by immuno-blotting with antibodies against FAM111B and α−Tubulin as a loading control. Clones negative for *FAM111B* expression were revived from liquid nitrogen and expanded.

### 4.7 Cell fractionation

Approximately 2 × 10^6^ of U2OS cells (either transiently transfected or not) was used for these experiments. Cells were washed once with ice cold PBS buffer, split in half (fractionation and whole lysate sets) and spun at 300 g for 3 min. Fractionation set was re-suspended in 100 μL of buffer A (10 mM HEPES pH7.4, 10 mM KCl, 1.5 mM MgCl2, 340 mM sucrose, 10% glycerol and 1 mM DTT) supplemented with protease and phosphatase inhibitors (Sigma). 2 μL of Triton X-100 (from 5% stock) was then added to the fractionation set, mixed for 3 s using vortex (highest speed) and placed on ice for 5 min. Lysates were then spun at 1300 g for 4 min at 4°C, 80 μL of the supernatant was collected as soluble fraction and mixed with 20 μL of 5× Laemmli buffer. Remaining pellet was gently washed with 1 mL of buffer A (pellet was not disturbed), spun again and re-suspended in buffer C (50 mM Tris-HCl pH7.4, 9 M Urea and 150 mM 2-mercaptoethanol), thoroughly sonicated and spun at 21 000 g. Similarly, 80 μL of the supernatant was collected as insoluble fraction and mixed with 20 μL of 5× Laemmli buffer. The whole lysate set was re-suspended in buffer C, sonicated and spun at 21 000 g for 15 min at 4°C. Again, 80 μL of the supernatant was collected as whole cell lysate sample and mixed with 20 μL of 5× Laemmli buffer. All samples were then boiled for 5 min at 95°C and analyzed by immuno-blotting (same volume of each sample was loaded) with the indicated antibodies.

### 4.8 C-circle assay

DNA was extracted from cells (10^6^ cells per sample) using the QIAGEN Core B kit and re-suspended in 20 mM Tris-HCl. 30 ng of genomic DNA was amplified in a PCR reaction containing φ29 polymerase, 1% Tween-20, 200 μg/mL BSA and dTTP, dGTP and dATP for 8 h at 30°C followed by 20 min at 65°C. PCR reactions were run with and without φ29 polymerase to ensure the signal was specific for rolling-circle amplification products. Amplified samples were then blotted onto Zeta-Probe membrane (Bio-Rad) using a slot blotter. DNA was crosslinked to the membrane with a UVA Stratalinker 2,400 and membranes were then soaked in PerfectHyb Plus (Sigma Aldrich) for 20 min at room temperature. A 3’ Digitonin (DIG) tagged [CCCATT]5 oligonucleotide was then diluted in PerfectHyb to a final concentration of 40 nM in 20 mL hybridization buffer, and was hybridized with the membrane for 2 h at 37°C. Following hybridization, membranes were briefly washed twice with wash buffer (0.1 M Maleic acid, 3 M NaCl; 0.1% Tween 20 adjusted to pH7.5), before blocking for 30 min and probing with a DIG antibody for 30 min. Finally, membranes were washed 3 times with wash buffer before placing into a cassette with CDP-Star solution. Blots were then developed onto Amersham Hyperfilm ECL film.

### 4.9 Growth curves and cell survival assay

For growth curves, MCF7 and U2OS cells were seeded in duplicate in 96 well plates at 2,500 cells per well (including no cells blank). Each day, separate duplicate wells were incubated with DMEM Fluorobrite Media (A1896701, GIBCO) supplemented with 10% fetal bovine serum, 1% glutamate, 1% penicillin/streptomycin and 10 μg/mL resazurin for 2 h. After the incubation time, fluorescence in the appropriate wells was read using microplate reader (CLARIOStarPlus, BMG LABTECH) with the following settings: excitation λ = 530 nm, emission λ = 590 nm. For the cell survival assays, 24 h post-seeding old media was replaced with fresh media containing the indicated drugs at different concentrations and cells incubated for another 72 h. On the final day the survival plates were assayed identically as growth curve plates. Cell growth was expressed as a relative % of wild-type cells whereas survival of the cells was expressed as relative % of the corresponding untreated samples.

### 4.10 Pull-down assays

For pull-down of FLAG-tagged proteins, transiently transfected HEK293T (single 10 cm dish per sample) were lysed in pull-down buffer (20 mM HEPES pH7.4, 150 mM KCl, 10% glycerol, 2 mM MgCl2, 0.5 mM DTT, 0.5% NP-40) supplemented with protease and phosphatase inhibitors (Sigma) as well as benzonase (250 U; Sigma) for 1 h at 4°C on a rotating wheel. Lysates were then spun at 21,000 g for 15 min at 4°C and protein concentration determined using Bradford assay (Sigma). Approximately 3–4 mg of total cell extract was mixed with 20 μL of M2-anti-FLAG agarose beads (bed volume) previously washed once with 1 mL of pull-down buffer and incubated for 2 h at 4°C on a rotating wheel. After the incubation, beads were spun (30 s at 2700 g at 4°C), washed 5 times with the pull-down buffer (at the second wash beads were transferred to a fresh tube to prevent contamination with sticky lysate proteins) and eluted with 30 μL of 3×FLAG peptide at 400 μg/mL for 30 min at 4°C shaking. Elutions were then mixed with 5× Laemmli buffer, boiled for 5 min at 95°C and analyzed by immuno-blotting with the indicated antibodies.

### 4.11 Flow cytometry

Approximately, 10^6^ of U2OS or MCF7 cells were pulsed with 50 μM BrdU for 5 min, harvested by trypsinization and re-suspended in 1 mL of PBS. 2 mL of −20°C absolute ethanol was then added dropwise to the cells while gently vortexing. Cells were fixed for at least 30 min at room temperature, spun for 5 min at 500 g and ethanol removed by two washes with PBS. To reveal BrdU epitopes, samples were treated with 1 mL of 2 M HCl/0.5% Triton X-100 solution for 30 min at room temperature, followed by acid neutralization with 1 mL of 0.1 M sodium tetraborate pH8.5. Cells were then blocked with 1% BSA/0.5% Triton X-100 in PBS for 30 min at room temperature, counted and brought to concentration of 10^6^ cells/mL. 20 μL of the anti-Brdu antibody was added to the cells and incubated for 1 h at room temperature, followed by three washes with 1% BSA/0.5% Triton X-100 in PBS and incubation in 100 μL of anti-mouse Alexa 488 in 1% BSA/0.5% Triton X-100 for 1 h at room temperature. Samples where again washed three times with 1% BSA/0.5% Triton X-100 in PBS and treated with RNase A at 100 μg/mL and propidium iodide at 40 μg/mL in PBS for 30 min at room temperature. Analysis of the samples was performed using SH800z Cell Sorter instrument (SONY).

### 4.12 Fluorescent *in-situ* hybridization

Chromosome spreads were prepared with standard techniques. Briefly, cells were incubated with Karyomax Colcemid (Thermo Fisher Scientific) 50 ng/mL for 3 h. Following a swelling step in hypotonic (Buffered Hypotonic Solution, Genial Helix), the cells were fixed twice in Carnoy’s fixative (methanol:acetic acid 3:1). The cells suspension was dropped onto clean slides, and air-dried.

The copy number and position of the FAM111B locus in the different cell lines was analyzed by FISH with fosmid G248P81352D4 (WI2-0409G07, hg19 chr11:58,859,240-58,898,785) mapping to FAM111B genomic position, and two BAC constructs flanking the region, RP11-100N3 (hg19, chr11:56481672-56641043) and RP11-286N22, hg19 chr11:61,197,622-61,249,870). The probes were labelled by nick translation, using a commercial kit (Abbott Molecular Nick Translation Kit) incorporating ChromaTide Alexa Fluor 488-5-dUTP (Thermo Fisher Scientific), ChromaTide Alexa Fluor 594-5-dUTP (Thermo Fisher Scientific), or biotin-16-dUTP (Sigma). The probes were re-suspended in hybridization buffer (50% formamide, 10% dextran sulfate, 2× SSC) at 10 ng/µL, in the presence of a 10× excess of unlabeled human Cot1 DNA (Sigma Aldrich). The cellular DNA was denatured in NaOH 0.07 M. The probe DNA was denatured by incubating it at 85°C and allowed to renature at 37°C for 30 min. The hybridization was carried out overnight at 37°C. Following post-hybridization washes in 0.1×SSC at 60°C, the biotinylated probe was visualized using Avidin Cy5 (ThermoFisher Scientific). A minimum of 25 images per cell line were acquired and analyzed with the Leica Cytovision software, on an Olympus) BX-51 epifluorescence microscope equipped with a JAI CVM4+ progressive-scan 24 fps black and white fluorescence CCD camera.

The TeloFISH was carried out using the Telomere PNA FISH Kit/Cy3 (Dako), following the manufacturer instructions. A minimum of 30 metaphases per cell line were acquired, using a Leica DM6B microscope for epifluorescence, equipped with a DFC 9000  Gt B&W fluorescence CCD camera, and operated via the Leica LASX software.

### 4.13 Mass spectrometry

Following the FLAG-IP, elutions were denatured in 200 μL 8 M urea (4.8 g/10 mL) in 100 mM TEAB for 30 min at room temperature, reduced with 10 mM TCEP, for 30 min at room temperature, alkylated with 50 mM iodoacetamide for 30 min at room temperature in the dark and finally diluted down to 1.5 M urea with 50 mM TEAB. Protein digestion was performed using 1.5 ng of trypsin in 50 mM TEAB over night at 37°C, followed by clean up with C18 column (PepMapC18; 300 µm × 5 mm, 5 µm particle size, Thermo Fischer) using solvent A (0.1% formic acid in water) at a pressure of 60bar and separated on an Ultimate 3000 UHPLC system (Thermo Fischer Scientific) coupled to a QExactive mass spectrometer (Thermo Fischer Scientific). The peptides were separated on an Easy Spray PepMap RSLC column (75 µm i.d. × 2 µm × 50 mm, 100 Å, Thermo Fisher) and then electro sprayed directly into an QExactive mass spectrometer (Thermo Fischer Scientific) through an EASY-Spray nano-electrospray ion source (Thermo Fischer Scientific) using a linear gradient (length: 60 min, 5%–35% solvent B (0.1% formic acid in acetonitrile and 5% dimethyl sulfoxide), flow rate: 250 nL/min). The raw data was acquired on the mass spectrometer in a data-dependent mode (DDA). Full scan MS spectra were acquired in the Orbitrap (scan range 380-1800 m/z, resolution 70000, AGC target 3e6, maximum injection time 100 m). After the MS scans, the 15 most intense peaks were selected for HCD fragmentation at 28% of normalized collision energy. HCD spectra were also acquired in the Orbitrap (resolution 17500, AGC target 1e5, maximum injection time 128 m) with first fixed mass at 100 m/z. Progenesis QI (Waters) based label-free quantitation results were imported into Perseus 1.5.2.463. Quantitative data was log2 transformed and normalized by median subtraction and missing values were imputed based on normal distribution.

### 4.14 Image and statistical analysis

Image analysis was performed using CellProfiler v. 4.2.1 software and home design pipelines. Briefly, for the measurements of TeloFISH foci intensities, individual chromosome objects were identified using Watershed algorithm and DAPI channel. Neighbouring nuclei were removed using twostep process: step one - Measure Object Size and step two Filter Objects by size (nuclei > chromosomes). Individual chromosomes were then used to mask TeloFISH channel (Mask Image) and telomers identified using Identify Primary Objects algorithm. Finally, intensity of individual TeloFISH signals was determined with Measure Object Intensity algorithm. For the TRF2 foci intensity measurements, nuclei were identified in DAPI channel (Identify Primary Objects) and used as a mask (Mask Image) to identify nuclear TRF2 objects coming from telomeres (Identify Primary Objects). The intensity of TRF2 objects was then measured using Measure Object Intensity algorithm. For FAM111B intensity in PCNA positive and negative cells, DAPI channel was used to identify nuclei which later masked PCNA channel. Intensity of PCNA in each nucleus was determined and a manual threshold was set up to differentiate PCNA positive and negative cells. Filtered PCNA nuclei were then masked onto FAM111B channel and FAM111B intensity quantified in both populations.

For the statistical calculations, Graphpad Prism 9 software was used. Briefly, normal distribution was assumed for experiments where manual counting was involved (Ona-Way Anova, OWA) whereas greater number of datapoints obtained with CellProfiler was analyzed with OWA (Gaussian distribution) or Kruskal–Wallis (K-W, non-Gaussian distribution) tests to determine whether the observed differences between sample groups were significant.

## Data Availability

The data will be made available immediately upon request. The original contributions presented in the study are included in the article/supplementary material, further inquiries can be directed to the corresponding author.
