# Supplementary material for: Loss of FAM111B protease mutated in hereditary fibrosing poikiloderma negatively regulates telomere length
Source: Front Cell Dev Biol. 2023 Jun 5;11:1175069. doi: 10.3389/fcell.2023.1175069 (PMC10277729; doi:10.3389/fcell.2023.1175069)
Supplement: Supplementary file 1 [file Table1.DOCX]

Supplementary Material

Loss of FAM111B protease mutated in hereditary fibrosing poikiloderma syndrome negatively regulates telomere length

Maciej Kliszczak^1*^, Daniela Moralli^1^, Julia D. Jankowska^1†^, Paulina Bryjka^1†^, Lamia Subha Meem^1†^, Tomas Goncalves^2^, Svenja S. Hester^3^, Roman Fischer^3^, David Clynes^2^ and Catherine M. Green^1^

*** Correspondence:** Maciej Kliszczak: maciej.kliszczak@ndm.ox.ac.uk

***Table 1. Primers used in this study***

| Name | Sequence | Purpose |
| --- | --- | --- |
| FAM111B_WT_FW_(N) | GGGGACAAGTTTGTACAAAAAAGCAGGCTTCAATTCCATGAAGACTGAAGAAAAC | N-terminal fusion |
| FAM111B_WT_RV_(N) | GGGGACCACTTTGTACAAGAAAGCTGGGTCC TAACATTCCATGGGTTCAATC |  |
| FAM111B_WT_FW_(C) | GGGGACAAGTTTGTACAAAAAAGCAGGCTTCA TGAATTCCATGAAGACTGAAGAA | C-terminal fusion |
| FAM111B_WT_RV_(C) | GGGGACCACTTTGTACAAGAAAGCTGGGTCAC ATTCCATGGGTTCAATCTG |  |
| FAM111B_H490A_FW | CCACCATAAGATGTACAACAGCTCGACAGGT GAAAATATAACCAC | N- and C-terminal fusions |
| FAM111B_H490A_RV | GTGGTTATATTTTCACCTGTCGAGCTGTTGTA CATCTTATGGTGG |  |
| FAM111B_Q430P_FW | GAAGTCCTTTTTATATATGTTGAATGGCTTAG CTGGTGAAAGATTCATTC | N- and C-terminal fusions |
| FAM111B_Q430P_RV | GAATGAATCTTTCACCAGCTAAGCCATTCAA CATATATAAAAAGGACTTCAAAAAGGACTTC |  |
| FAM111B_T625N_FW | TCTGATAGGAAACTTCTTTGGTTAAACATAC AGTATACATTACTGGT | N-terminal fusion  N-terminal fusion |
| FAM111B_T625N_RV | ACCAGTAATGTATACTGTATGTTTAACCAAA GAAGTTTCCTATCAGA |  |
| FAM111B_Δ279-386_FW | ACGTTTGATAATT CTTTAAGAGATCTTTCTGTT GTAATGCTTTTTTTTTTGAAATGTCC |  |
| FAM111B_Δ279-386_RV | GGACATTTCAAAAAAAAAAGCATTACAACAGAAAG ATCTCTTAAAGAATTATCAAACGT |  |
| gRNA_1_FW | CACCGGAAGGAGCCTTATGCAAGGA | *FAM111B* targeting |
| gRNA_1_RV | AAACTCCTTGCATAAGGCTCCTTCC |  |
| gRNA_2_FW | CACCGTCCAGATACTTCATCCACCA |  |
| gRNA_2_RV | AAACTGGTGGATGAAGTATCTGGAC |  |
