# Supplementary material for: Loss of FAM111B protease mutated in hereditary fibrosing poikiloderma negatively regulates telomere length
Source: Front Cell Dev Biol. 2023 Jun 5;11:1175069. doi: 10.3389/fcell.2023.1175069 (PMC10277729; doi:10.3389/fcell.2023.1175069)
Supplement: Supplementary file 4 [file DataSheet1.DOCX]

Supplementary Material

Loss of FAM111B protease mutated in hereditary fibrosing poikiloderma negatively regulates telomere length

Maciej Kliszczak^1*^, Daniela Moralli^1^, Julia D. Jankowska^1†^, Paulina Bryjka^1†^, Lamia Subha Meem^1†^, Tomas Goncalves^2^, Svenja S. Hester^3^, Roman Fischer^3^, David Clynes^2^ and Catherine M. Green^1^

*** Correspondence:** Maciej Kliszczak: maciej.kliszczak@ndm.ox.ac.uk


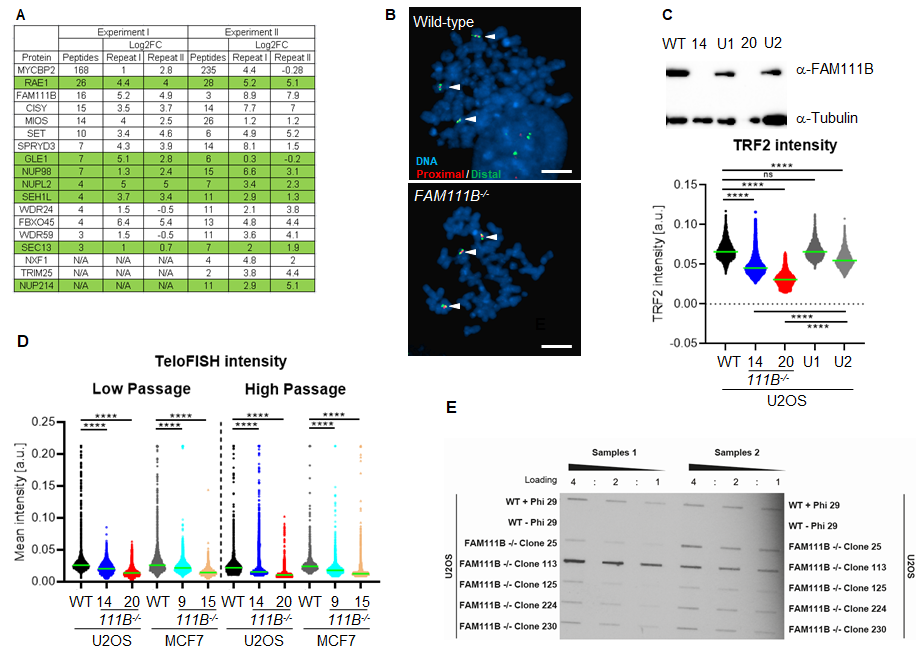

**Supplementary Figure 1. FAM111B interacts with the components of the nuclear pore complex. A)** Proteins detected in FLAG-FAM111B WT pull-downs. Table shows peptide number detected in each experiment and fold abundance over the FLAG control. **B)** Widefield images of chromosome spreads stained with fluorescent probes specific to *FAM111B* loci. Probes used in these experiments hybridize to regions flanking *FAM111B* gene (centromere proximal and distal probes). Intact patter of the probe indicates proper repair and lack of CRISPR induced rearrangements. Scale bar 5μm. **C)** Immuno-blots showing FAM111B levels in WT and different clones targeted with CRISPR-Cas9 in U2OS cells. Clone 14 and 20 are *FAM111B*-deficient clones whereas untargeted clones U1 and U2 have normal and lower levels of FAM111B, respectively (top panel). Quantification of the TRF2 intensity in the clones from top panel. Green bars show averages of N=3 experiments, with 400 cells scored per sample in each experiment. D) Quantification of TeloFISH intensity in *FAM111B*-defiecient U2OS and MCF7 cells from early and late passages. Green bars show averages of N=3 experiments, with at least 30 metaphase spreads scored per sample in each experiment. E) Southern slot blot showing levels of C-circles in multiple U2OS *FAM111B*-deficient clones. K-W test was used in C and D.


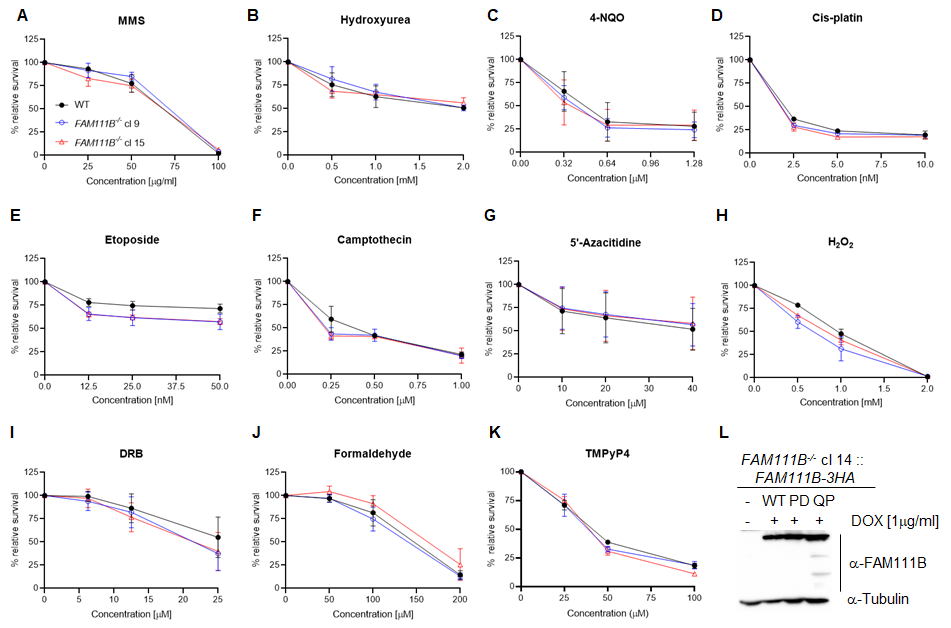
**Supplementary Figure 2. *FAM111B*-deficient cells show wild-type like response to DNA damage and DNA replication / transcription perturbations.** A) to K) survival of MCF7 wild-type (WT) and *FAM111B* knockout cells after chronic exposure to the indicated drugs. Error bars show standard error, N=3 experiments. L) Immuno-blot of U2OS *FAM111B^-/-^* cells in which FAM111B wild-type (WT), protease dead (PD) or HFP variant (Q430P) were re-introduced under the control of doxycycline (DOX) inducible promoter.


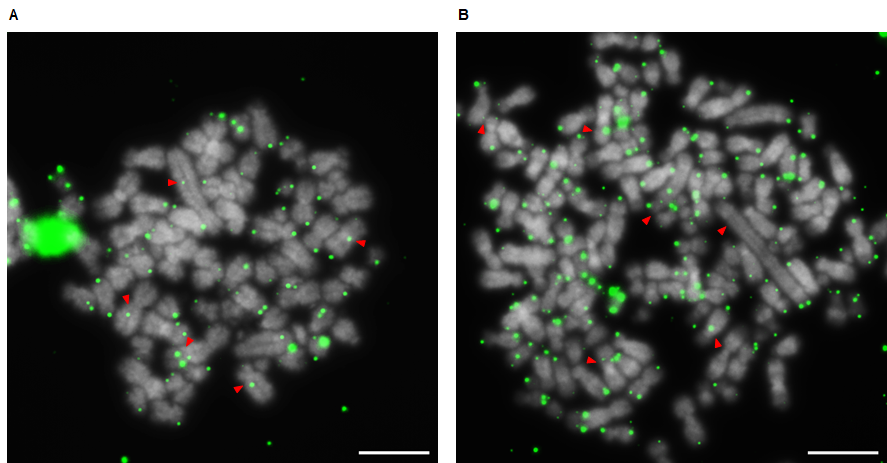


**Supplementary Figure 3. FAM111B deficient cells have increased number of telomeric fusions. A)** and **B)** Widefield images of metaphase spreads from *FAM111B*-deficient MCF7 (A) and (B) U2OS cells. Red arrowheads point towards fusion events or telomere fused chromosomes. Scale bar 5μm.

**Movie 1A. Localisation of FAM111B to the nuclear periphery.** Three-dimensional reconstitution of FAM111B staining in U2OS cells (DAPI staining DNA in Blue, antibodies staining of FAM111B in Green and Lamin A/C in Orange) U2OS cells. Movie shows different staining and masks:
Turn 1 - DNA | total FAM111B | Lamin A/C
Turn 2 - DNA
Turn 3 - Lamin A/C
Turn 4 - Total FAM111B
Turn 5 – DNA | Nuclear FAM111B
Turn 6 – DNA | Lamin A/C masked FAM111B
Turn 7 – Lamin A/C | Lamin A/C masked FAM111B.

**Movie 1B. Localisation of FAM111B to the nuclear periphery.** Three-dimensional reconstitution of FAM111B staining in U2OS cells (DAPI staining in Blue and antibodies staining of FAM111B in Green) showing only FAM111B signal masked with Lamin A/C (not shown).
